# Transformation of Amorphadiene Synthase and Antisilencing P19 Genes into *Artemisia annua* L. and its Effect on Antimalarial Artemisinin Production

**DOI:** 10.34172/apb.2020.057

**Published:** 2020-05-11

**Authors:** Elfahmi Elfahmi, Fany Mutia Cahyani, Tati Kristianti, Sony Suhandono

**Affiliations:** ^1^School of Pharmacy, Bandung Institute of Technology, Bandung, Indonesia.; ^2^Biosciences and Biotechnology Research Center, Bandung Institute of Technology, Bandung, Indonesia.; ^3^Institut Pendidikan Indonesia, Garut, West Java, Indonesia.; ^4^School of Life Sciences and Technology, Bandung Institute of Technology, Bandung, Indonesia.

**Keywords:** *Agrobacterium tumefaciens*, Artemisinin, *Amorphadiene synthase*, *Artemisia annua*, Malaria, p19

## Abstract

***Purpose:*** The low content of artemisinin related to the biosynthetic pathway is influenced by the role of certain enzymes in the formation of artemisinin. The regulation of genes involved in artemisinin biosynthesis through genetic engineering is a choice to enhance the content. This research aims to transform *ads* and *p19* gene as an antisilencing into *Artemisia annua* and to see their effects on artemisinin production.

***Methods:*** The presence of *p19* and *ads* genes was confirmed through polymerase chain reaction (PCR) products and sequencing analysis. The plasmids, which contain *ads* and/or *p19* genes, were transformed into *Agrobacterium tumefaciens*, and then inserted into leaves and hairy roots of A. annua by vacuum and syringe infiltration methods. The successful transformation was checked through the GUS histochemical test and the PCR analysis. Artemisinin levels were measured using HPLC.

***Results:*** The percentages of the blue area on leaves by using vacuum and syringe infiltration method and on hairy roots were up to 98, 92.55%, and 99.00% respectively. The *ads*-*p19* sample contained a higher level of artemisinin (0.18%) compared to other samples. Transformed hairy root with co-transformation of *ads*-*p19* contained 0.095% artemisinin, where no artemisinin was found in the control hairy root. The transformation of *ads* and *p19* genes into A. annua plant has been successfully done and could enhance the artemisinin content on the transformed leaves with *ads*-*p19* up to 2.57 folds compared to the untransformed leaves, while for *p19*, cotransformed and *ads* were up to 2.25, 1.29, and 1.14 folds respectively.

***Conclusion:*** Antisilencing *p19* gene could enhance the transformation efficiency of *ads* and artemisinin level in A. annua.

## Introduction

Malaria has been a serious problem in the world. In 2016, there have been more than 133 million people infected by *Plasmodium* spp, a malarial caused parasite, about 445 000 of them died.^1^ The use of antimalarial drugs, such as chloroquine, tends to be reduced because of drug resistance so that more effective drugs for malaria disease are needed.^2^ WHO has recommended the ACTs (artemisinin-based combined therapies) as a choice for treatment of malaria.^2,3^ Artemisinin, a sesquiterpene produced by *Artemisia annua* L. has an excellent effect on malaria in multi-drug resistant *Plasmodium* strains.^4,5^ Artemisinin together with its derivatives, especially dihydroartemisinin and artesunate, was reported to have good activity against *P. falciparum*.^5^

Recently, artemisinin has been reported to have potential effects for systemic lupus erythematosus. It could ameliorate renal damage, reduce the symptoms, and increase antibodies as well as proteinuria.^6^ To date, *A. annua* is the only source for artemisinin with a low yield.^7^ Because of its unique complex structure, the chemical synthesis is difficult, and it becomes less prospective. Other approaches to enhance the production of artemisinin are through cell culture and genetic engineering for the key enzymes of artemisinin biosynthesis in plant cell and yeast.^3,8,9^ Cell culture technique has advantages as an alternative system for recombinant pharmaceuticals.^8,10^ Farnesyl pyrophosphate is a precursor of artemisinin derivative biosynthesis. It is synthesized from one isoprenoid unit derived from the non-mevalonate pathway and two C-5 isoprenoid units derived from the mevalonate pathway in the cytosol.^9^ Farnesyl pyrophosphate is used by amorpha-4,11-diene synthase (*ads* ) as a precursor to produce cyclic amorpha-4,11-diene.^9,11,12^ Enzymes coded genes which have the key roles in the artemisinin biosynthesis have been cloned.^9,13^ Therefore, the enhancement of artemisinin production can be performed, using genetic engineering of these genes, and transform them into plants or microbes.^13^ Transient expression system of a gene in plants using agro-filtration has been developed as an alternative to optimize protein expression. Agro-infiltration has a flexible nature in the production of recombinant proteins in plant tissue and only need few days to get the results.^14-17^ Transient expression system with plant virus vector via *Agrobacterium* -mediated transformation has been performed for the production of recombinant protein with a high level and short time.^16-21^ The bacterium infects the plant cells and integrates a region of a large tumor-inducing (Ti) plasmid resident in *Agrobacterium* into the plant’s nuclear genome.^22^ An *Ads* gene-encoded *amorpha-4,11-diene synthase,* which is a key enzyme in artemisinin biosynthesis, has been transformed using vector pCAMBIA1303 resulting in plasmid pCAMBIA 1303-*ads.* The plasmid has been transformed into *A. annua*. *A. tumefaciens* strain AGL1, which is the most efficient transformation among others with up to 70.91% from the total explants of *A. annua* leaves.^23^ Although genetic transformation has been successfully done in plants, DNA of *A. tumefaciens* may activate the protection response in the plants, also called RNA silencing. Post-transcriptional gene silencing (PTGS) or RNA silencing is a natural protective response of plants from foreign nucleic acids, such as viral infection and transgene expression in plant cells, which can invade plants. In this process, the double-stranded, short-interfering RNA is cleaved from single-stranded RNA (ssRNA) and double-stranded RNA (dsRNA) or viral sequences by plant RNase III-type.^24-26^ The existence of PTGS will destroy the RNA of *A. tumefaciens* infected the plants so that the DNA transfer process in *A. tumefaciens* to the plants is not maximal. However, several plant viruses have the silencing suppressors which can inhibit the protection mechanism of plants. One of silencing suppressors is p19 gene from tomato bushy stunt virus.^27^ The purpose of this research is to evaluate the effect of a P19 gene in recombinant *A. annua* containing amorpha-4,11-diene synthase.

## Materials and Methods

There are a Luria-Bertani (LB) medium containing NaCl 1%, tripton 1%, *yeast* 0.5%, bacto agar 1.5%, and a liquid LB medium without bacto agar as a growing medium. TAE 1X {dilution from TAE 50X (Tris base 24.2% (Promega), acetic acid glacial 5.71 %, EDTA 0.5 M pH 8.0 10%)}; DNA 1 kb ladder (Fermentas^TM^); agarose (Top vision MDBio). DreamTaq Green MM (Fermentas^TM^), forward primers (5’-AAA CTC GAG ATG GAA CGA GCT ATA CAA G-3’), reverse primer (5’-AAA CTC GAG TTA CTC GCC TTC TTT TTC G-3’). Reagent for plasmid isolation containing solution 1 (glucose 50 mM, TrisCl 25 mM, EDTA pH 8 10mM, deionized up to 100 %), solution 2 (NaOH 0.2 N, SDS1 % w/v), and solution 3 (sodium acetate 5 M, acetic acid glacial), isopropanol, ethanol, and TE-RNAse.

### The preparation of p19 gene, plasmid pCAMBIA 1303 and pCAMBIA 1303- ads

*Ads* and synthetic *p19* genes were confirmed using polymerase chain reaction (PCR) with the composition of PCR reaction consisting of 12.5 µL of Dream Tag DNA polymerase, 1.25 µL of each forward and reverse primer as well as DNA template with the total volume of 25 µL after adding free-water nuclease. PCR products were detected using agarose gel 1.5% and then purified using a gel purification kit.

Plasmid pCAMBIA 1303 and pCAMBIA 1303-*ads* were cut by *Xho* 1 and incubated at 37°C for 9 minutes. Reactions were done at 37°C for 16 hours. Restriction results were checked by electrophoresis with agarose gel 1% for 30 min with 100 voltage. Bands with the size above 10 000 bp were purified. The *p19* gene whose sequence was confirmed was cut from pGEM-T Easy using Xho 1. Bands with a size of 527 bp were purified. Pure *p19* genes were measured of their concentrations and ready to be continued for transformation.

### The ligation of p19 gene into plasmid pCAMBIA 1303 and pCAMBIA 1303-ads 

*P19* gene was ligated into plasmid pCAMBIA 1303 and pCAMBIA 1303-*ads*, having been cut by *Xho* 1. The composition of ligation reaction consists of 1 μL of T4 DNA Ligase (0.03 unit), 1.6 µL of 10X Rapid ligation buffer, 6 µL of Plasmid CAMBIA 1303/pCAMBIA 1303-*ads* (24 ng), 4 µL of *p19* gene (16 ng) and nuclease free-water until the final concentration of 16 µL. Ligation was done at 4°C for 16 hours. Ligation reactions were transformed into *E. coli* DH5α in a LB medium containing kanamycin 50 ppm and incubated at 37°C overnight.

### Plasmid isolation

A single colony of transformants was selected and suspended in a liquid LB medium containing kanamycin 50 ppm and incubated in the shaker 200 rpm, 37°C overnight. Plasmids were collected and centrifuged to separate cell pellets. The pellets were re-suspended with buffer 300 μL then homogenized. The solution was centrifuged with 14 000 rpm for 5 minutes. 700 μL of supernatant was transferred to 1.5 mL microtube and added cold isopropanol (700 μL), then incubated with 14 000 rpm for 5 minutes. The supernatant was discarded. 40 μL of alcohol was added to the pellet and centrifuged for 5 minutes. Pellets were dried for 30-40 minutes, then re-dissolved with 30 μL TE buffer containing RNAse and incubated for 1 hour at 37°C. The solution was stored at -20°C for further analysis. Plasmid pCAMBIA 1303-p19 and pCAMBIA 1303-ads-p19 were confirmed by electrophoresis, PCR product analysis, restriction analysis, and sequencing.

### The transformation of pCAMBIA 1303-p19 and pCAMBIA 1303-ads-p19 into Agrobacterium tumefaciens and its confirmation 

Recombinant plasmids were transformed into *A. tumefaciens* AGL1. One hundred microliters of *A. tumefaciens* AGL1 was thawed and added 1 µg of plasmid, and then incubated on ice and liquid nitrogen for 5 minutes. Cells were incubated at 37°C for 25 minutes and added a liquid yeast-extract-peptone (YEP) medium 1 mL and incubated again at 37°C for 3 hours. Cells were centrifuged in 14 000 rpm for 1 minute. Pellets were re-suspended in 100 μL medium and inoculated in solid YEP containing ampicillin 100 ppm and kanamycin 50 ppm. A single colony was taken and suspended in liquid YEP medium containing ampicillin 100 ppm and kanamycin 50 ppm, and then incubated in a shaker at 250 rpm in the room temperature for 2x16 hours in a dark condition. The plasmid was isolated and characterized using electrophoresis 0.8% agarose and PCR product analysis with a specific primer of *p19* gene.

### The transformation of a plasmid containing A. tumefaciens into Artemisia annua L.

#### Vacuum infiltration method

Fourteen-day-old of *A. annua* was used for the explants. Leaves and hairy roots were incubated in 150 mL of MS basal medium for transformation mediated by *A. tumefaciens* AGL1. 10 mL of *A. tumefaciens* culture cells in YEP medium was grown until reached OD_600_ = 1. Pellets were re-suspended in 50 mL MS normal medium supplemented with acetosyringone and 0.002% surfactant Silwet S-408. The selected leaves were incubated in a MS medium and continued with the transformation of a plasmid containing *A. tumefaciens* into *A. annua* L. by vacuum infiltration method for 20 minutes at 8°C in a dark condition. Infected leaves and hairy roots were blotted using sterile filter paper and co-cultivated for 3 days in room temperature and a dark condition.

#### Syringe infiltration method

The selected leaves were incubated in a MS medium and continued with the transformation of plasmid-containing *A. tumefaciens* into *A. annua* L. with a syringe infiltration method in a dark condition. Syringe infiltration known as agroinfiltration, that is, when a gene is inserted into leaves transiently using a syringe without the needle. Infected leaves were blotted using with sterile filter paper and co-cultivated for 3 days in a room temperature and a dark condition.

#### The analysis of GUS transient expression

GUS transient expressions in *A. tumefaciens* infected explants were checked using histochemical method with 5-bromo-4-chloro-3-indolyl glucuronide (X-Gluc) staining.^28^ Each infected explant was rinsed in phosphate buffer pH 6.8 for 5 minutes and then incubated at 37°C for 24 hours in a dark condition. To strengthen the blue color, explants were washed with ethanol 70% until chlorophyll disappeared.^23^

#### The analysis of artemisinin content

Leaves of wild type, hairy roots, and transgenic of *A. annua* were dried and powdered, and then extracted with ethyl acetate 3x10 mL. Ethyl acetate was evaporated. The residue was dissolved with methanol 1.5 mL for derivatization. One hundred of methanol extract was added 400 μL of NaOH 0.05 N and then incubated for 30 minutes, continued with the addition of acetic acid 0.2 N, and then incubated once more on ice for 10 minutes. The methanol was added up to 1 mL, and then filtered with membrane filter 0.45 μm in size. The samples were injected to high-performance liquid chromatography (HPLC) system with Hewlett Packard Hwallet RP-18 (100 mm x 4.6 and particle size 5 μm) column. The mobile phase was the mixture of phosphate buffer (5 mM, pH 7): methanol: acetonitrile (60:30:10). The elution was gradient with flow of 0.6 mL/min, column temperature of 30°C. The detector was Diode Array Detector with 260 nm wavelength.^29^

## Results and Discussion

**Figure 1:**
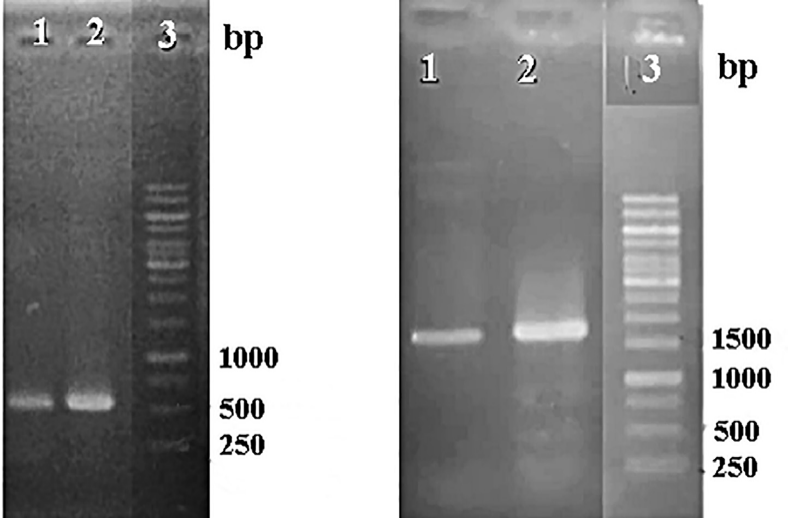
Electroforegram of PCR products of *p19* gene.1-2 (left) and ads gene from pCAMBIA-ads plasmid (right) 1-2; 3 = DNA ladder.

Synthetic *p19* gene from tomato bushy stunt virus which has a function in disturbing plant protection as PTGS has been amplified with PCR using DreamTaq Master Mix DNA Polymerase and a specific primer of *p19* gene. On the other hand, the*p19* gene is a virus gene which is easily mutated when it is amplified. Used primers were designed with extension for Xho restriction enzyme sites to be suitable for restriction analysis for both genes and pCAMBIA 1303. PCR results for confirmation of p19 from synthetic gene and ads gene from pCAMBIA-ads plasmid showed that both genes are detected in the right sizes (Figure 1). Electropherogram showed that band p19 has both size and intensity the same as the mass ruler DNA ladder 10 ng/μL. It is assumed that the concentration of the p19 gene is around 10 ng/μL. This p19 gene was further cloned into a cloning vector and transformed into *E. coli* DH5α. Furthermore, each p19 gene and pCAMBIA-ads plasmid was cut to prepare the cloning.

**Figure 2:**
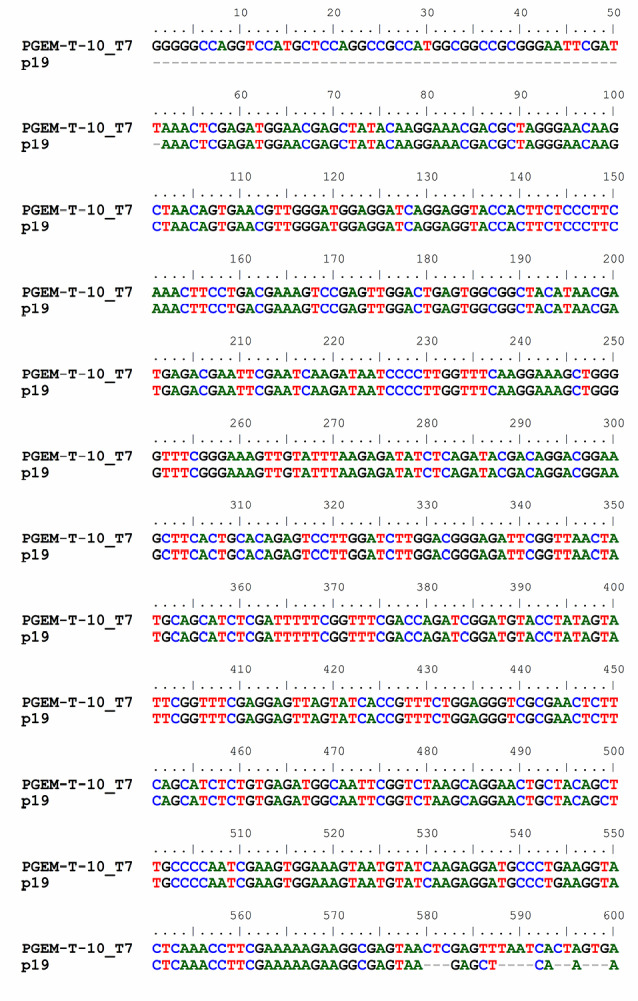
Sequence of p19 gene resulted from cloning with pGEM-T easy.

Ligation into cloning vector was done to prepare DNA insert in a large amount and the same condition. This was to minimalize the mutation event in the next ligation and to check whether the DNA insert in the right condition to be ready for cloning in an expression vector. Cloning is a process to duplicate the parent material, resulting in a large copy of genetic material which is completely the same as its parent material. To confirm the size of the DNA *p19* gene, the plasmid from a cloning result was cut by *Eco* R1 and *Xho* 1 enzyme. The result showed that the *p19* gene was successfully cloned into PGEM-T Easy. This has also been confirmed using sequencing analysis as shown in Figure 2.

**Figure 3:**
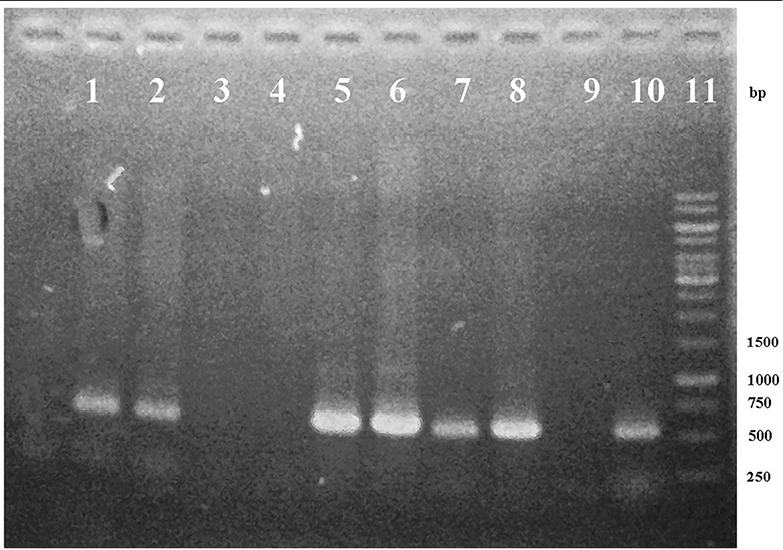
Electroforegram of PCR product of p19 gene from the genomic DNA of A. rhizogenes harboring pCAMBIA-P19 (1-2), wild type (3), pCAMBIA-ADS (4), pCAMBIA-ADS-P19 (5-6), positive controls (7-8), negative control (9), pCAMBIA-P19 (10) and DNA Ladder 1 kb (11).

Both pCAMBIA 1303 and pCAMBIA 1303-*ads* plasmids for ligation were prepared by discarding *hyg* gene (1000 bp) using *Xho* 1 enzyme. *Hyg* gene minus-plasmid was further purified and measured the concentration. The concentration of pCAMBIA 1303 and pCAMBIA 1303-*ads* plasmids after purification was 4 ng/μL each. *p19* gene was ligated into pCAMBIA 1303 and pCAMBIA 1303-*ads* plasmids. The availability of kanamycin resistance gene in a pCAMBIA 1303 vector could be used for the selection of transformants in colonies. To check whether the ligation reaction was successfully done or not, the results were analyzed by migration, PCR product, and restriction analysis. A single colony in solid LB medium was taken and re-suspended for isolation. From migration, restriction, and sequencing analysis, all of them showed that the p19 gene was successfully transformed to pCAMBIA 1303 vector, and pCAMBIA-ads plasmid resulted in pCAMBIA 1303-p19 and pCAMBIA-ads-p19 plasmids. Furthermore, both pCAMBIA-p19, pCAMBIA-ads-p19 and pCAMBIA 1303 were transformed into *A. tumefaciens* with a heat shock method. A successful transformation was checked by PCR using DNA templates from *A. tumefaciens* harboring each plasmid. PCR analysis results as in Figure 3 showed that the p19 gene could be detected in size 537 pb for pCAMBIA-p19 plasmid (lane 1, 2 and 10) and pCAMBIA-ads-p19 (lane 5 and 6). This band was also found in the positive controls (lane 7-8), while there were no bands in the same size found in *A. tumefaciens* wild type (lane 3), pCAMBIA-ads plasmid (lane 4) and negative control (lane 9). This confirmed that the p-19 gene in pCAMBIA 1303-p19 and pCAMBIA-ads-p19 were successfully transformed into *A. rhizogenes*.

**Figure 4:**
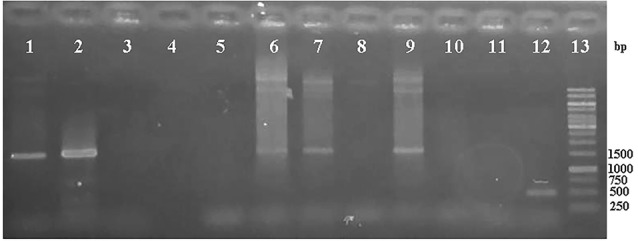
Electroforegram of PCR product of ADS gene from the genomic DNA of *A. tumefaciens* harboring pCAMBIA-ads-P19 (1-2), negative controls (3-5, 10), pCAMBIA-ads (6-7), wild type (8, 11), positive control (9), pCAMBIA-P19 (with primer p19) (12), DNA ladder 1kb (13).

No band in the *A. rhizogenes* wild type and harboring pCAMBIA-ads confirmed that p-19 gene did not come from the contaminated *A. tumefaciens* and pCAMBIA ads plasmid. To confirm the existence of ads gene in genomic DNA of *A. rhizogenes* harboring pCAMBIA-ads and pCAMBIA-ads-p19, PCR was done using ads primers. PCR analysis results, as shown in Figure 4, show that the ads gene could be detected in size 1500 bp for pCAMBIA-ads-p19 plasmid (lane 1,2), pCAMBIA-ads and positive control, while there were no bands in the same size found in negative controls (lane 3-5 and 10) and *A. tumefaciens* wild type (lane 8), indicating that the ads came only from the transformed plasmids.

Naturally, *A. tumefaciens* could transfer DNA fragment of its tumor-induced (Ti) plasmid into a plant genome to be expressed in plants.^30^ *A. tumefaciens* AGL1 as a carrier for recombinant plasmid that contained inserted gene was grown until reaching OD_600_ value 1. OD_600_ value 1 means the optimum condition where *A. tumefaciens* AGL1 can effectively be transformed. Two-week-old *A. annua* plant cell cultures were prepared to accept DNA insert of the p19 gene. The addition of surfactant will improve penetration into cuticule, so it will stimulate material transfer to plant cells. Surfactant Silwet S-408 has a significant effect in improving transformation efficiency in hairy root culture of *A. annua* up to 27.84 % out of the total infected explants.^23^

**Figure 5:**
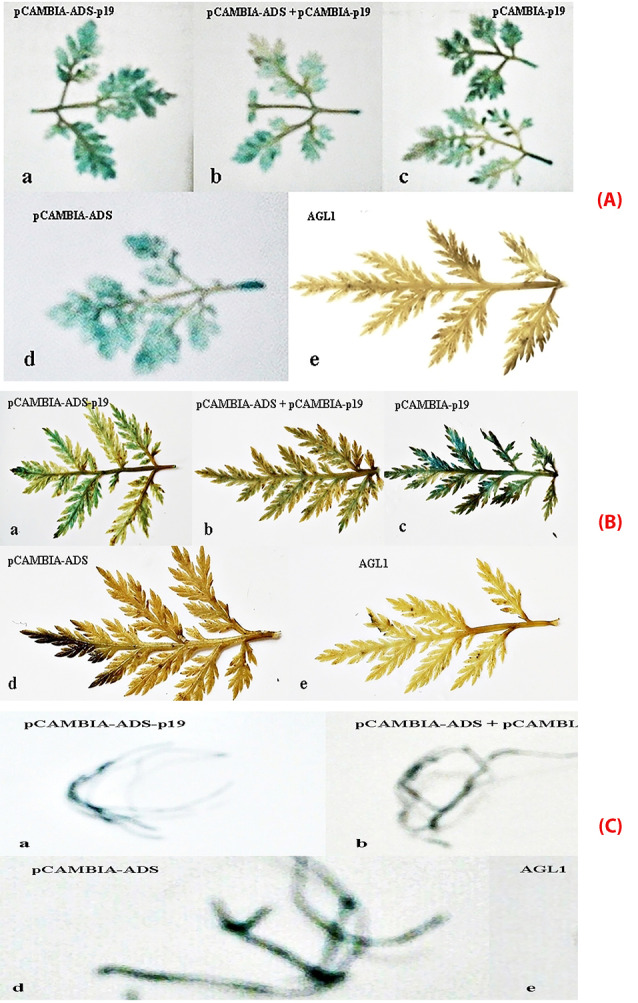
Transformed leaves of *Artemisia annua* L with *A. tumefaciens* AGL1 using vacuum (A), syringe (B), transformed hairy root (C) using vacuum infiltration methods containing (a) recombinant plasmid pCAMBIA 1303-ads-p19; (b) recombinant plasmid pCAMBIA 1303-co-transformation; (c) recombinant plasmid pCAMBIA 1303-p19; (d) recombinant plasmid pCAMBIA 1303-ads; (e) plasmid pCAMBIA 1303 (wildtype).

The transformation of *A. tumefaciens* AGL1 recombinants into *A. annua* was done using vacuum infiltration method for 20 minutes. Several research projects have proven this method could improve the expression frequency of *A. tumefaciens* into plants.^31,32^ Vacuum infiltration can exhaust the air in the intra cell so that the medium can enter the intra cell’s spaces. This method was also selected since it is easy to transfer genetic material to *A. annua* leaves with a small size where the syringe method is not suitable. The infected plants were furthermore incubated for 3 days to maximize the transient transformation into plants. The transformation was also done using syringe infiltration method. Syringe infiltration or agroinfiltration is a method used in plant biology to stimulate the expression of genes in a plant transiently to express the desired protein. This method is widely used technique to transform foreign genes into plant cells since it is simple, rapid, and versatile. The most popular method for agroinfiltration is syringe infiltration. This method is a simple procedure with no need for specialized equipment. A needleless syringe is used to apply *Agrobacterium* into plant leaves or other plant organs.^33^ In this method, the suspension of *A. tumefaciens* harboring the gene containing plasmids is infected into the plant leaves by direct injection. Furthermore, the bacteria transfer the inserted gene into the plant cells via T-DNA transfer. The benefit of the syringe infiltration method is not time-consuming and convenience. The yields of the recombinant protein are generally more consistent and much higher when compared to other traditional plant transformations. For the agroinfiltration method, Tween-20 could significantly improve the transformation efficiency with the optimal concentration of 0.03% (v/v).^33,34^ To check the transformation results which contain the *p19* gene with the plasmid pCAMBIA 1303 and plasmid pCAMBIA 1303-*ads*, histochemical method using GUS transient expression analysis was done. GUS gene is a gene attached to plasmid pCAMBIA and has an important role as reporting genes in genetic analysis. The expression of reporting gene could have the roles in several aspects, such as protein localization reporting,^35^ an indicator for translation activity, or a transduction signal, and successfully gene insert. Existence detection of β-glucuronidase (GUS) could be done qualitatively with histochemical GUS and quantitatively with spectrophotometric GUS.^28^ Based on histochemical test, the blue color appeared after adding substrate X-gluc (5-bromo-4-chloro-3-indolyl glucuronide). The percentage of blue area out of total area of leaves and hairy root showed that co-transformation pCAMBIA 1303-*ads* with pCAMBIA 1303-*p19* (Table 1, Figure 5) gave transformation efficiency value higher than direct transformation pCAMBIA 1303-*p19-ads* and the control pCAMBIA 1303-*ads*, while the transformation of *p19* gene gave the value higher than control pCAMBIA 1303. These results confirmed that the *ads* transformation could not optimally be expressed in *A. annua* due to the RNA silencing process by plant cells. *Ads* expression level was enhanced by co-transformation together with *p19* meaning that the *p19* gene suppressed the RNA silencing mechanism by plant cells. Other reports showed that *p19* gene of *Cymbidium* ring spot virus could inhibit RNA silencing through small RNA-binding activity. In the in vitro RNA-silencing system, small RNAs, bound by *p19* in plants, are double-stranded siRNAs and they are competent in silencing.^36^ During virus infection, *p19* could reduce the amount of free siRNA in cells through forming *p19* –siRNA complexes; therefore, siRNAs are inaccessible for effector complexes of RNA-silencing machinery. The *p19* -mediated sequestration of siRNAs in virus-infected cells inhibits the spread of the mobile, systemic signal of RNA silencing.^36^ The enhancement of the expression level was also shown by transformed hairy root with the antisilencing *p19* gene.

**Table 1:** Transformation efficiency of genes into *Artemisia annua* L. leaves using vacuum infiltration method (VIL), using syringe infiltration method (VSL) and hairy root using vacuum infiltration method

**Sample**	**Blue Area to Total Area (%)**		
	**VIL**	**VSL**	**HR**
WT	0	0	0
AGL1	0	0	0
*Ads*	58.47±1.27	39.04±9.01	80.21±6.38
*ads-p19*	97.13±1.2	92.55±1.39	96.00±0.26
*p19*	98.25±0.54	89.82±1.08	99.42±0.55
Co-T	85.92±0.13	65.22±6.87	91.17±0.79

**Note:**WT = wild type leaves, Transformed leaves with*Agrobacterium tumefaciens*, AGL1 = without plasmid and gene, *ads* = single plasmid with *ads* gene, *ads-p19* = single plasmid with *ads* and *p19* genes, *p19* = single plasmid with *p19* gene, Co-T = double plasmids, one with *ads*, another with p*19* gene.

### The analysis of artemisinin content using HPLC

The assay of artemisinin in samples was calculated by the equation lines derived from artemisinin standard calibration curve. The optimum conditions were obtained by using HP Hwallet column RP-18 (100 mm x 4.6 mm id, particle size 5 m), the mobile phase a mixture of phosphate buffer (pH 7, 5 mm) - methanol-acetonitrile (60:30:10, v/v) and a flow rate of 0.6 mL/min.^29^ Based on research conducted previously, it is stated that the column temperature is set at 30°C, which aims to improve measurement precision, improving separation, maintaining the retention time repeatability, sharpen chromatogram peak, increasing the efficiency of the column, as well as lowering the pump pressure.^29^

Based on the chromatogram of standard solution, artemisinin appeared at the fifth minute so that for the analysis of samples, it was focused on the fifth minute as well. The next step, the area is calculated in the linear regression equation. Chromatograms of the samples were compared with the control chromatogram. The controls were leaves without transformation and leaves that are transformed by AGL I-non containing genes (AGL I wildtype).

According to the obtained graphs, the hypothesis on the addition of the *p19* gene which can increase gene expression was proven, in this case, the gene *ads*. This is demonstrated by the samples that were transformed with *ads* gene only were not higher than the genes which were inserted simultaneously with *p19*. This means that the mechanism of PTGS actually occurs in *A. annua* plant and silencing suppressor *p19* suppresses the protection mechanism.

**Figure 6:**
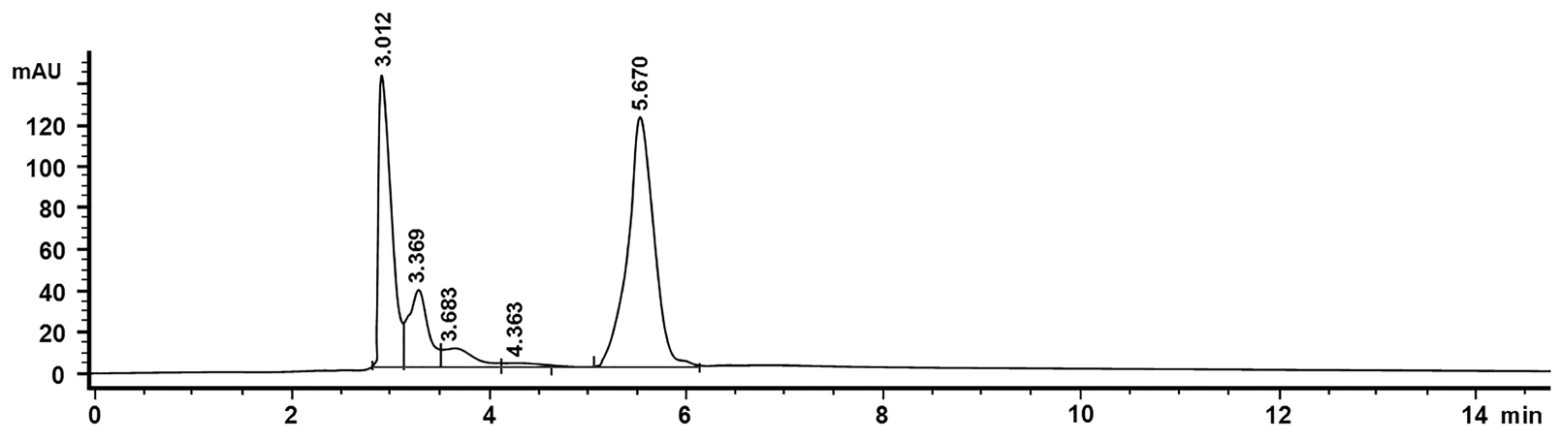
HPLC chromatogram of artemisinin (Rt 5.67 min) with 20 ul injection volume. The flow rate of 0.6 ml / min. Phosphate buffer mobile phase: methanol: acetonitrile (60:30:10).

**Figure 7:**
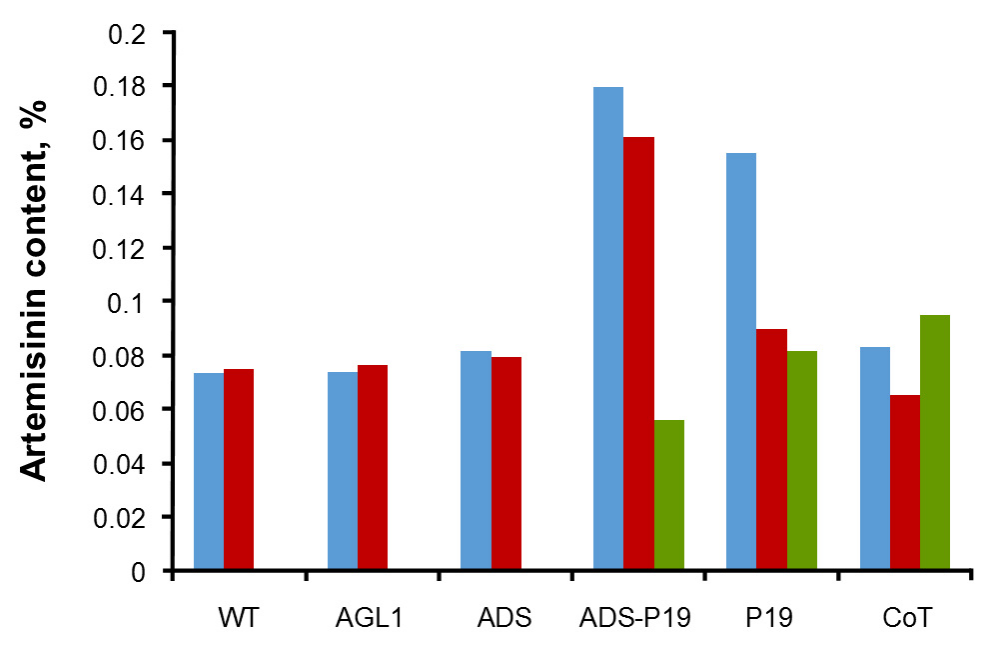
The artemisinin content of Artemisia annua leaves wildtype (wt) and Agrobacterium tumefaciens-mediated transgenic leaves and hairy root (blue bars = VIL, red bars = VSL and green bars = HR) without inserted gene (AGL 1), with inserted ads gene (ADS), with inserted ads and p19 genes in single plasmid (ADS-P19), with inserted p19 gene (p19), with inserted ads and p19 genes with different plasmid (CoT).

The transformation of plasmids containing genes *ads*, *ads-p19*, *p19* and co-transformation have been successfully performed on wildtype leaves and hairy root of *A. annua*, based on histochemical GUS and artemisinin content analysis using HPLC with chromatogram, as shown in Figure 6. The levels of artemisinin derived from analysis by HPLC for samples without transformation, agli, *ads*, *ads-p19*, *p19* and co-transformation using vacuum infiltration method were 0.07, 0.074, 0.08, 0.18, 0.16, and 0.083% respectively, while using syringe infiltration method were 0.07, 0.07, 0.08, 0.17, 0.09, and 0.07% (Figure 7). For the hairy root culture, the co transformation of *ads* and *p19* in each plasmid could produce artemisinin 0.095%, while no artemisinin was found in the untransformed hairy root. It can be concluded that the *ads-p19* gene using vacuum infiltration method could increase the artemisinin compound in *A . annua* wildtype compared with single *ads* gene plasmid or single *p19* gene plasmid.

## Conclusion

Amorpha-4,11-diene synthase (*ads* ), a key enzyme of antimalarial artemisinin, has been transformed in *A. annua* leaves, mediated by *A. tumefaciens*. In addition, the antisilencing of thep19 gene has also been transformed into this plant to increase the expression level of *ads* gene. The transformation of *ads* and *p19* genes into leaves of *A. annua* has enhanced the artemisinin content on transformed leaves with *ads-p19* up to 2.57 folds compared to untransformed leaves, while for *p19*, co-transformed and *ads* were up to 2.25, 1.29, and 1.14 folds respectively.

## Ethical Issues

Not applicable.

## Conflict of Interest

The authors confirm that this article content has no conflicts of interest.

## Acknowledgments

The author would like to thank the Directorate of Research and Community services, Ministry of Research, Technology and Higher Education, Republic of Indonesia, and Bandung Institute of Technology for the financial support for the research.

## References

[1] World Health Organization (WHO). World Malaria Report 2017. Geneva: WHO; 2017.

[2] van Herpen TW, Cankar K, Nogueira M, Bosch D, Bouwmeester HJ, Beekwilder J (2010). Nicotiana benthamiana as a production platform for artemisinin precursors. PLoS One.

[3] Paddon CJ, Westfall PJ, Pitera DJ, Benjamin K, Fisher K, McPhee D (2013). High-level semi-synthetic production of the potent antimalarial artemisinin. Nature.

[4] Winstanley PA, Ward SA, Snow RW (2002). Clinical status and implications of antimalarial drug resistance. Microbes Infect.

[5] Fairhurst RM, Dondorp AM (2016). Artemisinin‐resistant Plasmodium falciparum malaria. Microbiol Spectr.

[6] Mu X, Wang C (2018). Artemisinins-a promising new treatment for systemic lupus erythematosus: a descriptive review. Curr Rheumatol Rep.

[7] Khosla C, Keasling JD (2003). Metabolic engineering for drug discovery and development. Nat Rev Drug Discov.

[8] George EF, Sherrington PD. Plant Propagation by Tissue Culture. England: Exegetics Limited; 1984.

[9] Muangphrom P, Seki H, Fukushima EO, Muranaka T (2016). Artemisinin-based antimalarial research: application of biotechnology to the production of artemisinin, its mode of action, and the mechanism of resistance of Plasmodium parasites. J Nat Med.

[10] Xu J, Zhang N (2014). On the way to commercializing plant cell culture platform for biopharmaceuticals: present status and prospect. Pharm Bioprocess.

[11] Kim SH, Heo K, Chang YJ, Park SH, Rhee SK, Kim SU (2006). Cyclization mechanism of amorpha-4,11-diene synthase, a key enzyme in artemisinin biosynthesis. J Nat Prod.

[12] Picaud S, Mercke P, He X, Sterner O, Brodelius M, Cane DE (2006). Amorpha-4,11-diene synthase: mechanism and stereochemistry of the enzymatic cyclization of farnesyl diphosphate. Arch Biochem Biophys.

[13] Covello PS, Teoh KH, Polichuk DR, Reed DW, Nowak G (2007). Functional genomics and the biosynthesis of artemisinin. Phytochemistry.

[14] Kapila J, De Rycke R, Van Montagu M, Angenon G (1997). An Agrobacterium-mediated transient gene expression system for intact leaves. Plant Sci.

[15] Van der Hoorn RA, Laurent F, Roth R, De Wit PJ (2000). Agroinfiltration is a versatile tool that facilitates comparative analyses of Avr9/Cf-9-induced and Avr4/Cf-4-induced necrosis. Mol Plant Microbe Interact.

[16] Lindbo JA (2007). TRBO: a high-efficiency tobacco mosaic virus RNA-based overexpression vector. Plant Physiol.

[17] Sainsbury F, Varennes-Jutras P, Goulet MC, D’Aoust MA, Michaud D (2013). Tomato cystatin SlCYS8 as a stabilizing fusion partner for human serpin expression in plants. Plant Biotechnol J.

[18] Turpen TH, Turpen AM, Weinzettl N, Kumagai MH, Dawson WO (1993). Transfection of whole plants from wounds inoculated with Agrobacterium tumefaciens containing cDNA of tobacco mosaic virus. J Virol Methods.

[19] Escobar MA, Dandekar AM (2003). Agrobacterium tumefaciens as an agent of disease. Trends Plant Sci.

[20] Musiychuk K, Stephenson N, Bi H, Farrance CE, Orozovic G, Brodelius M (2007). A launch vector for the production of vaccine antigens in plants. Influenza Other Respir Viruses.

[21] Robert S, Khalf M, Goulet MC, D’Aoust MA, Sainsbury F, Michaud D (2013). Protection of recombinant mammalian antibodies from development-dependent proteolysis in leaves of Nicotiana benthamiana. PLoS One.

[22] Gelvin SB (2003). Agrobacterium-mediated plant transformation: the biology behind the “gene-jockeying” tool. Microbiol Mol Biol Rev.

[23] Elfahmi Elfahmi, Suhandono S, Chahyadi A (2014). Optimization of genetic transformation of Artemisia annua L Using Agrobacterium for artemisinin production. Pharmacogn Mag.

[24] Huang TK, Falk BW, Dandekar AM, McDonald KA (2018). Enhancement of recombinant protein production in transgenic Nicotiana benthamiana plant cell suspension cultures with co-cultivation of Agrobacterium containing silencing suppressors. Int J Mol Sci.

[25] Voinnet O (2001). RNA silencing as a plant immune system against viruses. Trends Genet.

[26] Trinks D, Rajeswaran R, Shivaprasad PV, Akbergenov R, Oakeley EJ, Veluthambi K (2005). Suppression of RNA silencing by a geminivirus nuclear protein, AC2, correlates with transactivation of host genes. J Virol.

[27] Voinnet O, Rivas S, Mestre P, Baulcombe D (2003). An enhanced transient expression system in plants based on suppression of gene silencing by the p19 protein of tomato bushy stunt virus. Plant J.

[28] Jefferson RA (1987). Assaying chimeric genes in plants: the GUS gene fusion system. Plant Mol Biol Report.

[29] Fajrina S (2012). High pressure liquid chromatography method for analysis of artemisinin from Artemisia annua in vitro cultures [thesis]. Bandung Institute of Technology.

[30] Tzfira T, Citovsky V (2006). Agrobacterium-mediated genetic transformation of plants: biology and biotechnology. Curr Opin Biotechnol.

[31] de Oliveira ML, Febres VJ, Costa MG, Moore GA, Otoni WC (2009). High-efficiency Agrobacterium-mediated transformation of citrus via sonication and vacuum infiltration. Plant Cell Rep.

[32] Li S, Cong Y, Liu Y, Wang T, Shuai Q, Chen N (2017). Optimization of Agrobacterium-mediated transformation in soybean. Front Plant Sci.

[33] Zhao H, Tan Z, Wen X, Wang Y (2017). An improved syringe agroinfiltration protocol to enhance transformation efficiency by combinative use of 5-azacytidine, ascorbate acid and tween-20. Plants (Basel).

[34] Zhang J, Yu D, Zhang Y, Liu K, Xu K, Zhang F (2017). Vacuum and co-cultivation agroinfiltration of (germinated) seeds results in tobacco rattle virus (TRV) mediated whole-plant virus-induced gene silencing (VIGS) in wheat and maize. Front Plant Sci.

[35] Clark DP, Pazdernik NJ. Molecular Biology: Understanding the Genetic Revolution. 2nd ed. San Diego: Elsevier/Academic Press; 2012.

[36] Lakatos L, Szittya G, Silhavy D, Burgyan J (2004). Molecular mechanism of RNA silencing suppression mediated by p19 protein of tombusviruses. EMBO J.

